# Evolutionary trajectory of phenological escape in a flowering plant: Mechanistic insights from bidirectional avoidance of butterfly egg‐laying pressure

**DOI:** 10.1002/ece3.11330

**Published:** 2024-04-29

**Authors:** W. James Davies, Ilik J. Saccheri

**Affiliations:** ^1^ Institute of Infection, Veterinary and Ecological Sciences, Department of Evolution, Ecology and Behaviour University of Liverpool Liverpool UK

**Keywords:** *Anthocharis cardamines*, *Cardamine pratensis*, disrupted flowering schedule, evolutionary trajectory, phenological escape, pre‐dispersal seed predation

## Abstract

Phenological escape, whereby species alter the timing of life‐history events to avoid seasonal antagonists, is usually analyzed either as a potential evolutionary outcome given current selection coefficients, or as a realized outcome in response to known enemies. We here gain mechanistic insights into the evolutionary trajectory of phenological escape in the brassicaceous herb *Cardamine pratensis*, by comparing the flowering schedules of two sympatric ecotypes in different stages of a disruptive response to egg‐laying pressure imposed by the pierid butterfly *Anthocharis cardamines*, whose larvae are pre‐dispersal seed predators (reducing realized fecundity by ~70%). When the focal point of highest intensity selection (peak egg‐laying) occurs early in the flowering schedule, selection for late flowering dependent on reduced egg‐laying combined with selection for early flowering dependent on reduced predator survival results in a symmetrical bimodal flowering curve; when the focal point occurs late, an asymmetrical flowering curve results with a large early flowering mode due to selection for reduced egg‐laying augmented by selection for infested plants to outrun larval development and dehisce prior to seed‐pod consumption. Unequal selection pressures on high and low fecundity ramets, due to asynchronous flowering and morphologically targeted (size‐dependent) egg‐laying, constrain phenological escape, with bimodal flowering evolving primarily in response to disruptive selection on high fecundity phenotypes. These results emphasize the importance of analyzing variation in selection coefficients among morphological phenotypes over the entire flowering schedule to predict how populations will evolve in response to altered phenologies resulting from climate change.

## Graphical abstract

We gain mechanistic insights into the evolution of phenological escape, whereby organisms alter the timing of life history events to avoid antagonists, by comparing the flowering schedules of two sympatric ecotypes of the same plant species in early and late phases of a disrupted phenological response to butterfly egg‐laying pressure. We provide evidence that plant morphology, anti‐predator defense mechanisms and flowering time interact to determine the availability of alternative flowering refugia and hence direct the evolutionary trajectory of phenological escape.

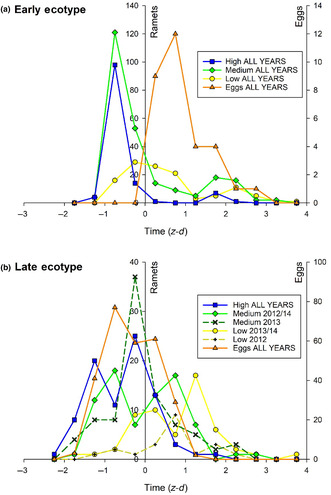

## 1 INTRODUCTION

Reproductive timing is a critical life history trait, particularly in species responding to seasonal environments, where the optimal reproductive window is finite and may be of short duration (Rafferty et al., 2015). For angiosperms, flowering time is subject to strong environmental and ecological constraints. Abiotic factors, such as temperature and rainfall, exert ‘bottom‐up’ selection pressures on plants to flower in environments within their physiological tolerance range, whereas biotic agents exert ‘top‐down’ pressures on plants to flower synchronously with the appearance of mutualists, such as pollinators and seed dispersers, and asynchronously with the activities of antagonists, such as herbivores and pre‐dispersal seed predators (Elzinga et al., 2007). Spatio‐temporal variation in the overall strength and direction of selection on flowering phenology is ubiquitous (Ehrlén, 1996; Elzinga et al., 2007; Kolb et al., 2007; Munguia‐Rosas et al., 2011). Selection on flowering phenology may interact with selection on correlated traits (Ehrlén, 2015), such as plant size (Munguia‐Rosas et al., 2011); the evolutionary response to trade‐offs arising from genetic covariance may render the flowering time of the otherwise fittest phenotypes sub‐optimal or even maladaptive (Lande, 1979; Lande & Arnold, 1983).

The relative strength of various selection pressures acting on phenology may change through the flowering period (Ehrlén et al., 2015; Pettersson, 1994) so that early flowering plants are exposed to a different selective regime than late flowering ones. Selection pressures operating outside the flowering window may also affect flowering time, emphasizing that phenological events are the outcome of ontogenetic processes integrated within a life history context (Ehrlén, 2015; Inouye et al., 2019). The rapidity with which plants can respond to phenological selection pressures (Chuine, 2010; Colautti & Barrett, 2013; Franks et al., 2007) favor the evolution of locally adapted populations within complex, dynamically shifting adaptive landscapes (Hereford, 2009); coevolutionary feedbacks with mutualists and antagonists shape the formation of geographical selection mosaics (Kolb et al., 2007; Thompson, 2005).

Phenological shifts in key life history events are ubiquitous responses among living organisms to climate change (Parmesan, 2006). However, the magnitude and direction of these shifts vary among taxa, with the potential to decouple critically important interactions through emergent phenological mismatches (Rafferty et al., 2015). Changes in flowering phenology are unlikely to be equal to shifts in the appearance and activity of mutualists and antagonists (Kőrösi et al., 2018), altering the phenotypic selection gradients to which plants are exposed. Demographic outcomes due to mismatches (Miller‐Rushing et al., 2010), as well as those due to new phenological alignments (Kiers et al., 2010; Nakazawa & Doi, 2012), will further alter the adaptive landscape (Davies, 2019). To accurately predict ecological and evolutionary outcomes of unequal phenological responses to climate change, a mechanistic understanding of how organisms respond to multiple interacting selection pressures is required. Such an understanding can be sought in the evolutionary trajectory of existing responses to phenological pressures, which can be unraveled if separate demes in different phases of a similar response to the same selective regime are available for comparison.

**FIGURE 1:**
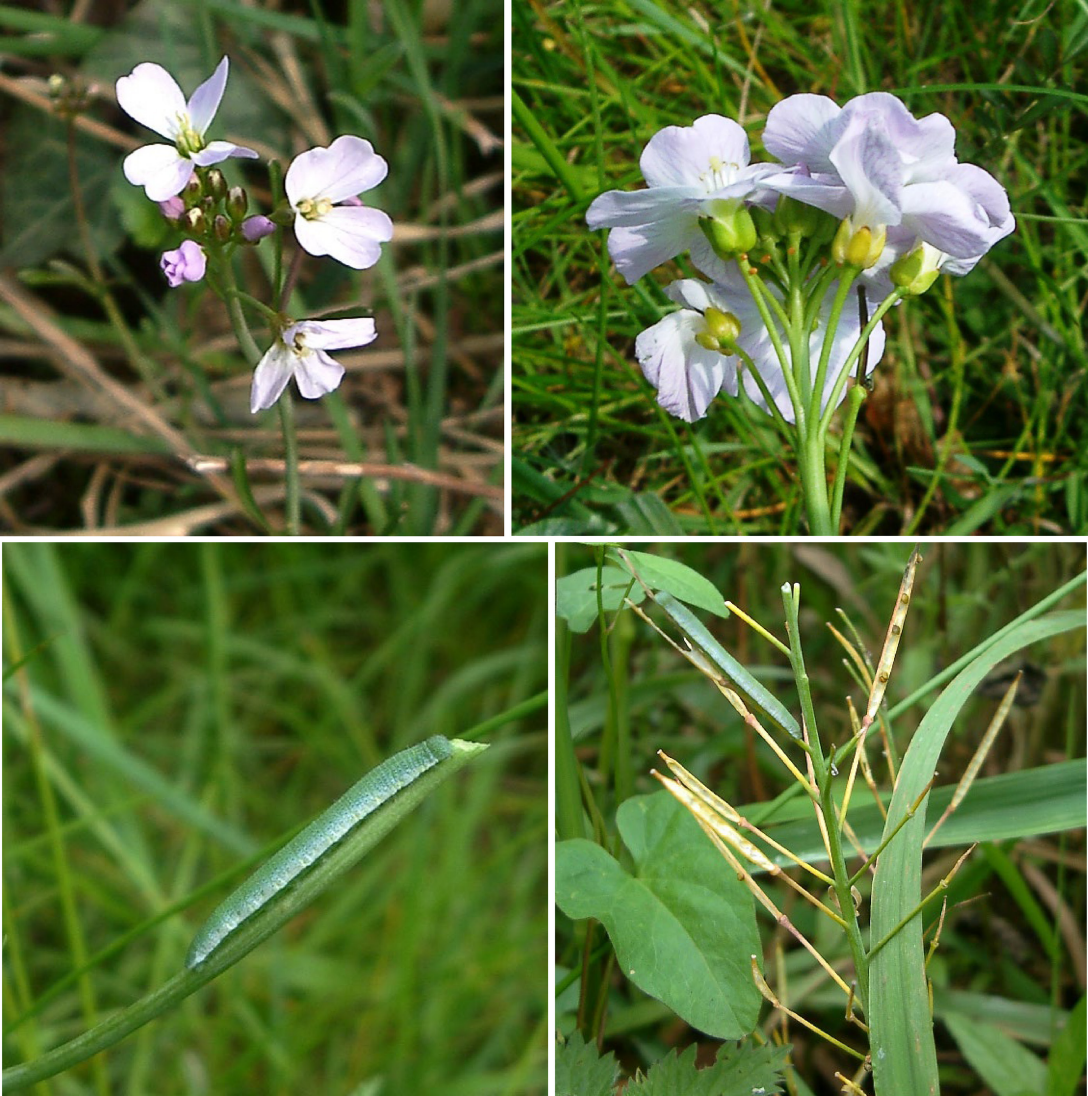
[Top] *Cardamine pratensis* “early” (left) and “late” (right) ecotypes (the latter with six orange *Anthocharis cardamines* eggs below the central calyces). [Bottom] (left) *A. cardamines* final instar larva on the axial stem of a late ecotype plant. The upper part of the stem has been wholly consumed, and no seed‐pods remain. (right) Final instar larva on an early ecotype plant, which has progressed to dehiscence before the larva could consume the seed pods.

The antagonistic interaction between the brassicaceous herb *Cardamine pratensis* and the pierid butterfly *Anthocharis cardamines* offers wide scope for the evolution of phenological avoidance. *C. pratensis* is a perennial that reproduces both sexually and vegetatively (stoloniferous ramification). It can therefore be regarded as a super‐organism formed by the plants (ramets) in each clonal colony (genet). The perennial and vegetative reproductive strategies maximize seed production during the lifetime of each genet, increasing the chances of colonization of new sites, which is dependent on long‐range seed dispersal and essential for the reproductive success of the super‐organism. *A. cardamines* larvae are pre‐dispersal seed‐predators (Duggan, 1985) that frequently consume the entire aerial portion of the plant when feeding on *C. pratensis* (Figure 1). The flowering period of *C. pratensis* (~12 weeks) is about twice as long as the flight period of the butterfly (~6 weeks); *C. pratensis* usually begins to flower in advance of the emergence of *A. cardamines* (Sparks & Yates, 1997), but it has been observed to commence flowering later (Dennis & Hardy, 2006). Hence, the plant has access to two phenological refugia on either side of the appearance of a butterfly, which has the potential to impose significant losses on seed production.

Selection on phenology may be difficult to disentangle from selection on plant size if the two traits are correlated (Ehrlén, 2015; Munguia‐Rosas et al., 2011). There is a weak trend in the literature for plant size to be negatively correlated with flowering time (Munguia‐Rosas et al., 2011). The need for resource accumulation prior to flowering is predicted to limit the ability of smaller plants within a population to flower early (Munguia‐Rosas et al., 2011), an effect which may be particularly marked in perennials, with larger (presumably older) plants able to flower earlier due to the accumulation of resources in previous seasons (Forrest & Miller‐Rushing, 2010). This has the potential to modify the phenological interaction with pre‐dispersal seed predators if they preferentially target plants in a particular size category (Kolb et al., 2007), or if fitness losses among different sized plants are unequal (Ollerton & Lack, 1998). *A. cardamines* females preferentially oviposit on *C. pratensis* plants with large shoot size (Arvanitis et al., 2008; Dennis & Hardy, 2006) or large flower heads (Dempster, 1997); since these flower earlier at least in some populations (Dempster, 1997), there is the potential for an interaction between plant size and phenology in any evolutionary response of the perennial to the butterfly.

In a recent study in Sweden, early flowering *C. pratensis* plants were more heavily attacked by egg‐laying *A. cardamines* females in two out of the 4 years in the study period, with no effect of phenology in the other 2 years; larger plants were preferentially selected for oviposition in all 4 years (König et al., 2015). The greater vulnerability of earlier flowering plants corroborated previous results from the same locality (Arvanitis et al., 2008); though intermittent, phenotypic selection against early flowering is likely persistent. However, no information is yet available as to whether plant phenology is responding to this selection pressure or whether it is interacting with selection on plant size. In a broad scale study utilizing data collected over the entire United Kingdom, Phillimore et al. (2012) found no evidence for a coevolutionary interaction between the phenologies of *A. cardamines* and *C. pratensis*. However, these conclusions are based solely on citizen scientist observations of dates of first appearance of the target species, whereas a more detailed investigation over the entire flowering schedule may be required to detect an evolutionary signal in their interaction.

The ancient coevolutionary interaction (~80 myr) between brassicales and pierine butterflies has been dominated by the appearance and escalation of innovations relating to the production of glucosinolates by the plants and a detoxifying nitrile specifier protein by the butterflies (Edger et al., 2015). Shortly after the appearance of the Brassicaceae 32 mya, two clades within the Pierinae, the Pierina (to which the cabbage white (*Pieris*) butterflies belong) and the Anthocharidinae (to which *A. cardamines* belongs), independently colonized them as host‐plants (Edger et al., 2015). Hence, whereas glucosinolates are excellent deterrents and poisons of generalist herbivores, they act as attractants to pierids. In consequence, members of the Brassicaceae have evolved a suite of defense mechanisms specifically to cope with pierid attack, including egg killing (Fatouros et al., 2014), retardation of larval growth (Pashalidou et al., 2013), and attraction of parasitoids through infochemical (volatile) release (Fatouros et al., 2014). The possible existence of these mechanisms has not yet been investigated for the interaction between *C. pratensis* and *A. cardamines*, but Courtney (1981) suggested that older plants present mechanical barriers (hardened siliquae) to grazing which prevents establishment of larvae on them (see also Wiklund & Åhrberg, 1978). The presence of such defenses could weaken the pressure for predator avoidance, and hence impact the evolution of phenological escape.

Here, we take advantage of the fact that two sympatric ecotypes of *C. pratensis* in a locality in NW England exhibit contrasting phenological interactions with *A. cardamines*. Since one ecotype exhibits a disrupted flowering schedule and largely avoids oviposition by flowering on either side of the butterfly's flight period, it is close to phenological escape. The other ecotype flowers synchronously with *A. cardamines* and is heavily subjected to egg‐laying by it, but exhibits a flowering curve consistent with the early phases of the evolution of a disrupted response. Specifically, we aim to:

1. test the validity of the assumption that *A. cardamines* is driving these responses;
2. investigate the interactions between ramet size, flowering date and egg‐laying behavior to accurately characterize phenotype‐dependent selection pressures operating on plant phenology;
3. identify any anti‐predator defense mechanisms that modify these selection pressures;
4. model the predicted phenological fitness landscape to see if bimodal flowering is favored;
5. gain mechanistic insights into the evolutionary trajectory of phenological escape.

## 2 METHODS

### 2.1 *C. pratensis* ecotypes

The two *C. pratensis* ecotypes (hereafter “early” and “late”) are distinguished by flowering time, morphology (Figure 1) and habitat. The early ecotype flowers earlier, is smaller, has more non‐axial inflorescences (“side‐branches”) and inhabits wetter areas than the late ecotype. Full phenological and morphological characteristics of the two ecotypes during the study period are given in Table S1.

### 2.2 Study site

Dibbinsdale Nature Reserve is located on the Wirral peninsula in NW England (for a map see Davies & Saccheri, 2013). It comprises 475 ha semi‐natural ancient woodland running parallel to Dibbinsdale Brook. The early ecotype occurs abundantly along the river bank and in damp places generally; the late ecotype is restricted to a single dry field situated on high ground. *A. cardamines* occurs abundantly in the Reserve; its adult population size is ~300 (both sexes) in most years (Davies & Saccheri, 2015). We surveyed the flowering and egg‐laying patterns for the two ecotypes over a 3 year period (2012–14). In all, 302 eggs were found on 810 hosts; 31 eggs on 31 early ecotype plants out of 487 checked, and 271 eggs on 152 late ecotype plants out of 323 examined.

### 2.3 Host‐plant transects and butterfly flight season

Transects on which every ramet was kept under observation from first flowering date until dehiscence were selected at suitable places in the Reserve (away from public pathways to avoid flower‐picking and where an abundance of pre‐flowering plants had previously been observed) and revisited every 5–7 days. Newly flowering plants were individually labeled and the number of buds, flowers, seed‐pods, newly laid eggs, old eggs and 1st to 5th instar larvae were recorded for each plant on every visit. For the late ecotype, eggs were sometimes laid on pre‐flowering plants; these were labeled and kept under observation until they were formally added to the transect at first flowering date.

The flight season of *A. cardamines* females was determined from dates of capture and recapture of all specimens encountered in the Reserve. Box‐and‐whiskers plots show the median, 25th/75th percentiles (edge of box), 10th/90th percentiles (whiskers) and outliers (circles) of the capture + recapture data in a given season.

### 2.4 Data analysis

Flowering curves (number of newly flowering plants versus date) were plotted for three fecundity classes within each ecotype. We define the fecundity of the ramets to be the total number of buds + flowers + seed‐pods (reproductive units, RU) counted on the date at which their sum maximized (usually on or shortly after first flowering date). For each year in the study period, we define “high”, “medium”, and “low” fecundity classes to include ramets in the upper quartile, interquartile, and lower quartile range of fecundity values, respectively (up to a slight excess/shortfall in some classes due to plants with borderline fecundity values being assigned to one of them only). Hence, there are twice as many medium fecundity ramets as there are high or low fecundity ones. This procedure was adopted since a more even numerical spread diluted important phenological effects in the outer two classes. The exact demarcation line between fecundity classes varied with the average fecundity of the plants among years, removing environmental effects on plant growth in different seasons.

The bimodality in flowering date of the early ecotype is obvious from inspection of its flowering curve, since the two modes are strongly defined and well separated temporally. For the more continuous flowering curve of the late ecotype, there is a possibility of confusion between true multi‐modality and random fluctuation in flowering density. To standardize interpretation of these curves, we define a flowering distribution to be bimodal only if it has two peaks separated by two or more intervening data‐points whose mean is below the value of either peak. Hence, the two flowering peaks must be separated by at least 2 weeks, and there must be a well defined trough between them. Bimodality was also assessed by combining flowering curves across years using *z*‐score transformed data (units of SD from mean flowering date in each year) to standardize flowering time. For analyses in which the flowering curve was split into two portions around each mode, for the early ecotype the dividing line is defined by the reappearance of larger plants in the general population, since this is indicative of a new wave of flowering; for the late ecotype the dividing line is defined by the appearance of the first flowering plants after the low point in the inter‐modal trough of the flowering curve.

### 2.5 Mathematical model

We calculate the fitness, *F*, of ramets with *R* reproductive units and phenology *z* (*z*‐score flowering day), as

$$F\left(R,z\right)=R.\left[I^{-}\left(R\right).f^{-}\left(R,z\right)+I^{+}\left(R\right).f^{+}\left(R,z\right)\right]$$

where $I^{-}$ = percentage *R* intact at dehiscence in absence of 5th instar larvae, $f^{-}$ = frequency of plants with 5th instar larvae absent, $I^{+}$ = percentage *R* intact at dehiscence in presence of 5th instar larvae, $f^{+}$ = frequency of plants with 5th instar larvae present; *F* and *f* are functions of both *R* and *z*; *I* is a function of *R* only. Now

$$f^{+}\left(R,z\right)=E\left(R,z\right).L$$

$$f^{-}\left(R,z\right)=1-E\left(R,z\right).L$$

where *E*(*R, z*) = egg load on plants (a function of *R* and *z*), *L* = proportion of eggs producing 5th instar larvae (independent of *R* and *z*). We shall here confine our attention to fitness at peak flowering date z*. Hence

(1) $$F\left(R,z^{*}\right)=R.\left(I^{-}\left(R\right)-E\left(R,z^{*}\right).L.\left[I^{-}\left(R\right)-I^{+}\left(R\right)\right]\right)$$

*E*(*R*, *z*) is modeled as a Gaussian function (see Figure 6):

$$E\left(R,z\right)=a\left(R\right).e^{-\frac{1}{2}{\left(\frac{z-z_{0}\left(R\right)}{b\left(R\right)}\right)}^{2}}$$

with coefficients *a*(*R*), *z*_*0*_(*R*) and *b*(*R*) for each fecundity class *R* given in the footnote to Figure 6 (since *z*_*0*_ is negative, it has changed the sign in the equations given there). Equation 1 gives fitness as a function of both fecundity and phenology. We also calculate fitness as a function of phenology only by summing over the individual fitnesses of each fecundity class at the time of peak flowering for that class z*(*R*) to obtain:

(2) $$F\left(z^{*}\right)=\sum_{R}p\left(R\right).R.\left(I^{-}\left(R\right)-E\left(R,z^{*}\left(R\right)\right).L.\left[I^{-}\left(R\right)-I^{+}(R\right]\right)$$

where *p*(*R*) = proportion of plants in fecundity class *R*. Equations 1 and 2 calculate fitness for individual and combined fecundity classes, respectively, based on the alternative assumptions that flowering time will evolve independently for each class, or as a weighted response to selection acting on all classes. In both cases, we compute the fitness for early and late season flowering peaks and subtract the fitness at the mean date between them to calculate the change in fitness due to disruptive selection, Δ*F*, and hence the Relative Fitness Gain = Δ*F*/*F*.

## 3 RESULTS

### 3.1 Phenological and morphological characteristics of *C. pratensis* early and late ecotypes

The phenological and morphological characteristics of the two *C. pratensis* ecotypes over the study period are summarized in Table S1. The late ecotype flowered later (~25 days) but developed to dehiscence faster (by ~8 days) than the early ecotype. Late ecotype ramets were taller (~35% increase in maximum height) and bore fewer non‐axial inflorescences than early ecotype ramets, suggesting that the latter compensate for small size by producing more side‐branches. These differences were significant; there were also trends for the late ecotype to be taller at first flowering and slightly more fecund (~1.3 more RU) than the early ecotype. There was no difference in flowering duration between the two ecotypes.

### 3.2 Interaction of *C. pratensis* flowering with *A. cardamines* egg‐laying

**FIGURE 2:**
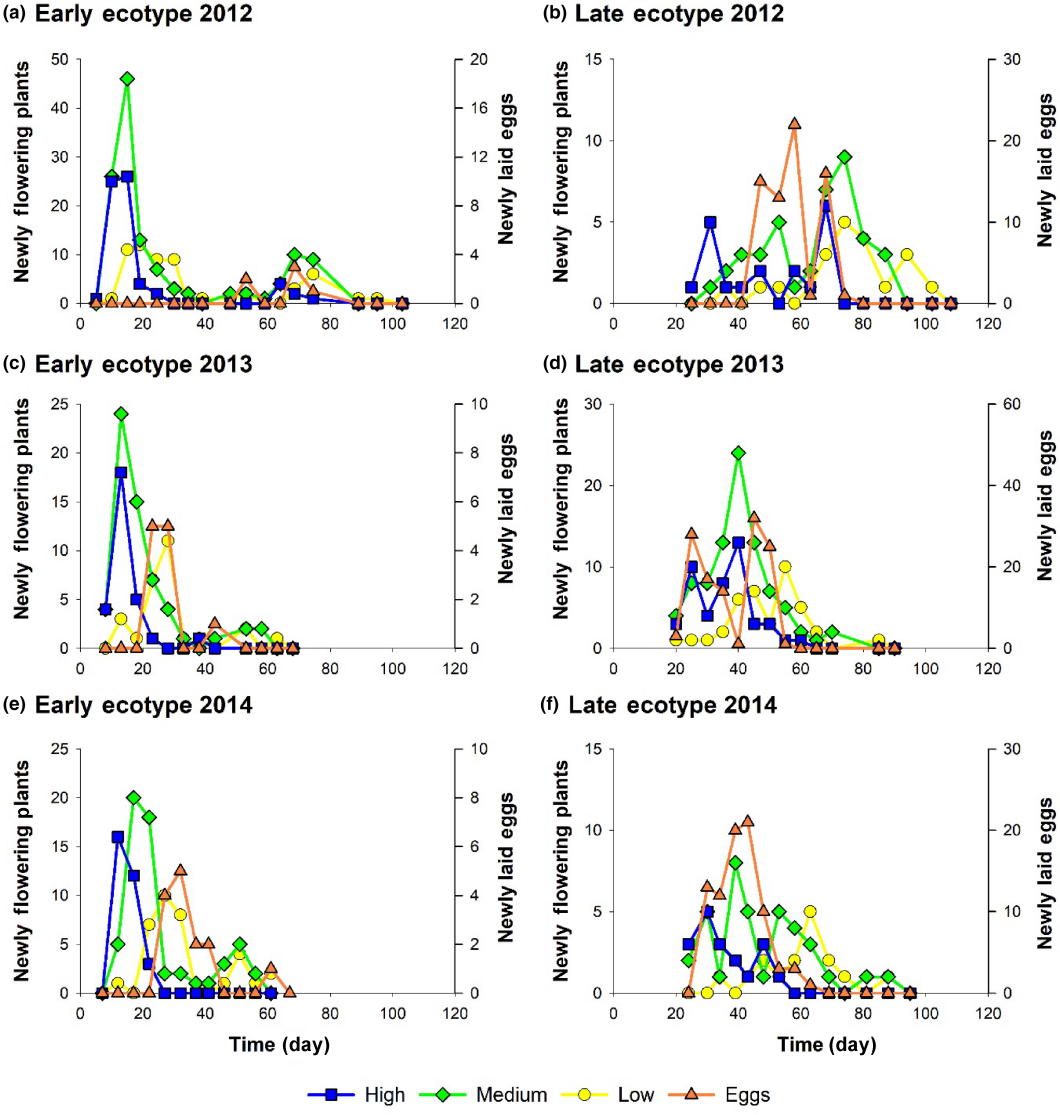
Flowering curves of high, medium and low fecundity ramets of the early (a, c, e) and late (b, d, f) *Cardamine pratensis* ecotypes with the corresponding egg‐laying curves of *Anthocharis cardamines* in Dibbinsdale Nature Reserve 2012–2014. The absence of an egg‐laying peak for the early ecotype in 2012 (a) was due to suppression of oviposition in early season by inclement weather. Day 1 is the date on which the first early ecotype ramet was observed to flower in the Reserve; graphs are therefore phenologically aligned between years.

**FIGURE 3:**
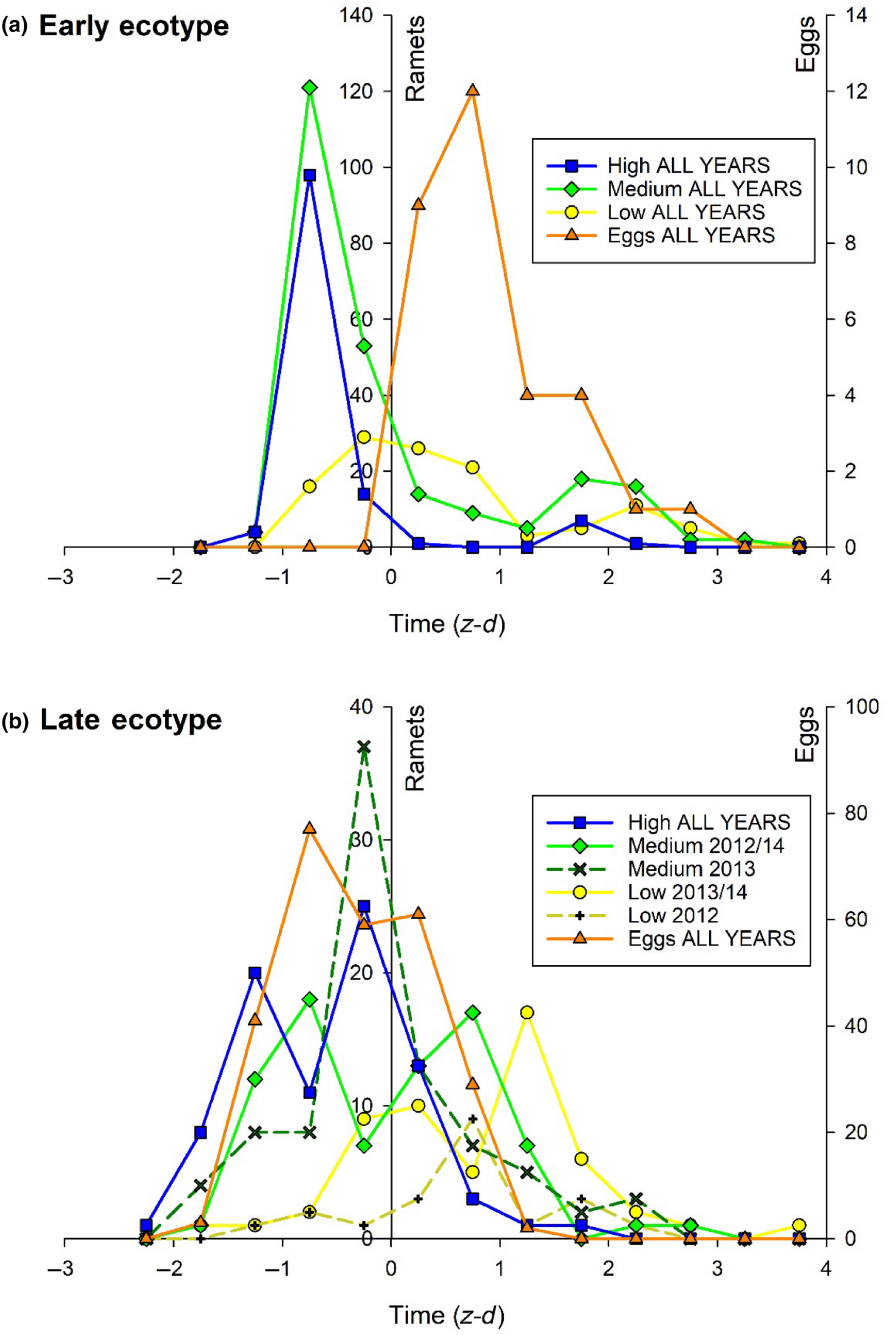
Aggregated flowering curves for high, medium and low fecundity ramets together with egg‐laying curves for the early and late *Cardamine pratensis* ecotypes. Data in different years have been standardized by converting date to a *z*‐score (*z*‐*d* = units of SD from mean flowering date in each year).

**FIGURE 4:**
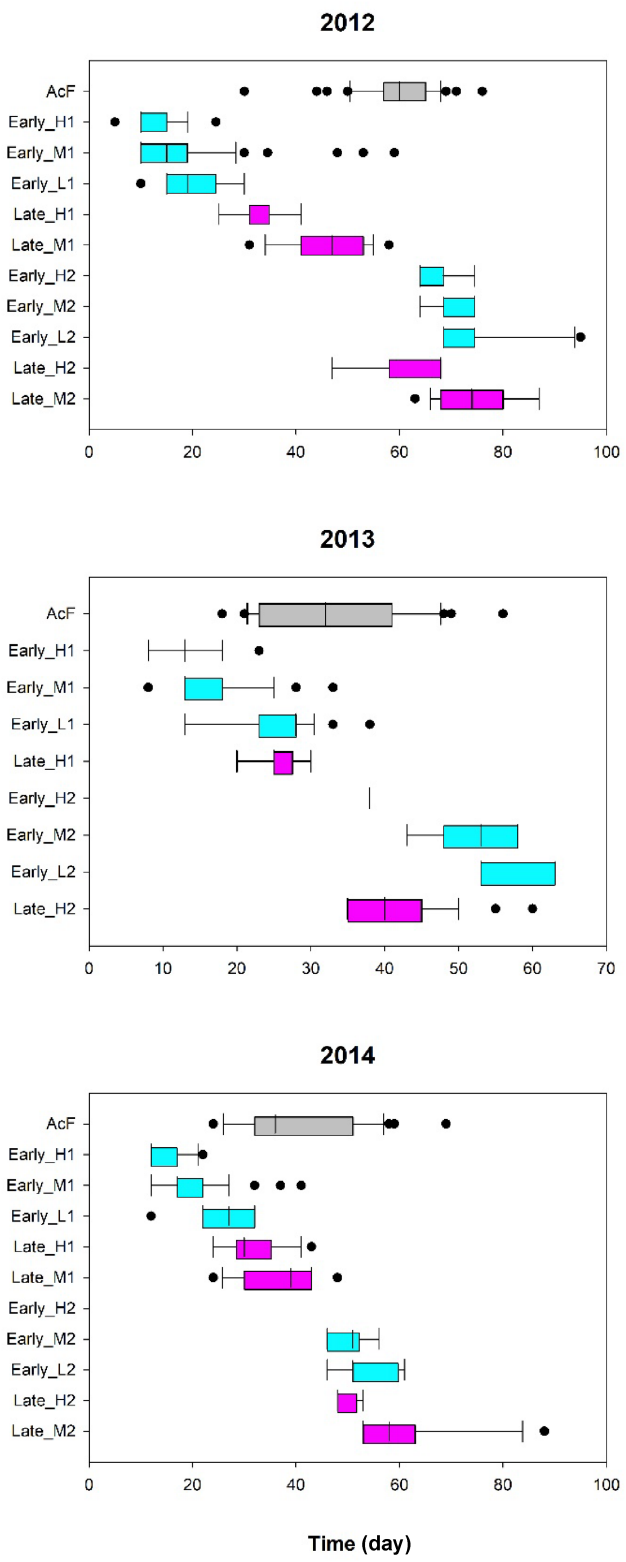
Relative flight period of *Anthocharis cardamines* females (AcF) and flowering period of *Cardamine pratensis* high (H), medium (M) and low (L) fecundity ramets around the first (1) and second (2) modes of the bimodal flowering curves for the early and late ecotypes. There were no E_H2 ramets in 2014, and only 1 in 2013. The L_M flowering curve was not bimodal in 2013 (see Figure 2d). Day 1 is the date on which the first early ecotype ramet was observed to flower in Dibbinsdale Nature Reserve; graphs are therefore phenologically aligned between years.

The two ecotypes exhibited flowering curves consistent with early and late phases in the evolution of phenological avoidance of egg‐laying *A. cardamines* females. The early ecotype has progressed further towards phenological escape. The flowering curve is bimodal with the early season mode much larger than the late season one (Figures 2a–e and 3); the flight season of *A. cardamines* females extends between the two modes (Figure 4). Around each mode, flowering time advances with increasing fecundity (Figures 2, 3, 4). Hence, the high fecundity ramets are phenologically furthest from *A. cardamines* females in early season but closest to them in late season (Figure 4). However, high fecundity ramets are rare in late season for this ecotype (Figure 2a,c), and were absent in 2014 (Figure 2e). Peak egg‐laying was consistently situated between the early and late season modes of the high and medium fecundity ramets (Figures 2c,e and 3), demonstrating phenological avoidance, but was coincident with the early season mode of the low fecundity ramets (which are rarely attacked by females) in 2013 (Figure 2c). These data imply that fecundity‐biased oviposition pressure of *A. cardamines* females is the requisite disruptive selective agent responsible for the evolution of the bimodal flowering curve.

The late ecotype exhibits incomplete phenological avoidance. The flowering curve of the high fecundity ramets was consistently bimodal (Figures 2b,d,f and 3) and that of the medium fecundity ramets was bimodal in 2012 and 2014, with early and late season peaks lagging behind those of the high fecundity ramets (Figures 2b,f and 3), producing an inverse relationship between fecundity and flowering date (Figures 3 and 4), but unimodal in 2013 (Figures 2d and 3). Bimodality could not be clearly distinguished in the flowering curves of the low fecundity ramets in individual seasons, but the combined curves for 2013 and 2014 did exhibit bimodality, with the two peaks lagging behind those of the medium fecundity ramets (Figure 3), extending the inverse relationship between fecundity and flowering date over all three classes. Hence, a clear and consistent bimodal response is only produced in high fecundity ramets; it is clear but inconsistent in medium fecundity ramets, and neither clear nor consistent in low fecundity ramets. In years when bimodality was absent, peak flowering occurred between the two peaks of the standardized bimodal curve for other seasons (Figure 3). The flight season of *A. cardamines* females extends between the two modes (when present), but with greater overlap than occurs for the corresponding modes of the early ecotype (Figure 4); peak egg‐laying was usually situated between the modes of the high fecundity ramets (Figures 2b,f and 3). These data suggest that bimodal flowering is in process of evolving from a unimodal curve, and is currently best canalized in high fecundity ramets.

### 3.3 Oviposition behavior and fitness cost to plants

If egg‐laying behavior of *A. cardamines* has been responsible for the evolution of a disrupted flowering curve in *C. pratensis*, then females should select newly flowering plants for oviposition (so that flowering time matters) and the resulting larvae should inflict a high fitness cost on them. Since the phenological response is stronger in high fecundity ramets, the selection of/damage to these plants should be correspondingly greater. However, since phenological escape is further advanced in the early ecotype, its flowering curve is also predicted to offer increased protection against the behavior of the butterfly.

The average time elapsed (±SE) between first flowering date and egg‐laying date on the early ecotype was 11.50 ± 1.44 days (*N* = 31); however, a strong peak in egg‐laying near first flowering date (Figure S1a) indicates that females prefer newly flowering plants but are either unable to find them efficiently or such plants are too scarce at the time of egg‐laying due to their precocious phenology. This conclusion is supported by the strong egg‐laying peak coincident with first flowering date and the shorter 5.72 ± 0.53 day (*N* = 270) average interval between flowering and egg‐laying on the more phenologically exposed late ecotype; interestingly, 11% of eggs were actually laid on plants before first flowering (Figure S1b). Females also showed a strong propensity to oviposit on higher fecundity ramets of this ecotype (Figure S2b) since oviposition preference is unmodified by the phenology of the plants. For the early ecotype, a similar tendency of females to select high fecundity plants is counterbalanced by the fact that they were no longer newly flowering (and hence favored for oviposition) at the time of peak egg‐laying; in this case, the advanced phenology of the most vulnerable phenotypes successfully suppresses oviposition (Figure S2a).

Full‐grown (final instar) *A. cardamines* larvae inflict a high fitness cost on *C. pratensis* (Table 1). In many cases, the plants are entirely consumed (Figure 1). For the late ecotype, the number of reproductive units (RU) intact at dehiscence was lower in high and medium fecundity plants subjected to larval grazing than in ungrazed plants; in both cases, the proportionate reduction in realized fecundity attributable to larvae was significant (Table 1). The average effect of larval infestation across all fecundity classes was to lower seed‐pod production by ~70% (Table 1) in spite of the fact that, due to the oviposition preference of *A. cardamines* females, infested plants started out with a significantly higher (*z* = 3.69, *p* = .0002) potential fecundity. Only three full‐grown larvae resulted from eggs laid on the early ecotype transects. Further data were therefore obtained on the effects of grazing on this ecotype in 2017 and 2018 (Figure S3a). Of those plants bearing final instar larvae, 41% were wholly consumed before dehiscence. Interestingly, 34% dehisced before the larva completed its development, indicating that the plants can outrun the larvae and disperse seeds before consumption (Figure 1). This outcome is clearly more likely when egg‐laying is delayed, as is usual on this ecotype (Figure S1a) due to the advanced flowering of the plants; on the late ecotype, where egg‐laying is ~6 days earlier on average and may even precede flowering (Figure S1b), only 4% of plants dehisced in advance of the completion of larval development, and 58% were wholly consumed (Figure S3b). These differences are significant ($\chi_{2}^{2}$ = 11.95, *p* = .003).

**TABLE 1:** Mean values (±SE) of the maximum number of reproductive units (buds + flowers + seed‐pods) prior to dehiscence (a measure of potential fecundity) and the intact number at dehiscence for late ecotype ramets in each fecundity class subjected to grazing by final instar *Anthocharis cardamines* larvae (+L) or not (−L).

Fecundity class	Category	N	Max. R.U.	Intact R.U.	% Intact	Grazing cost	$\chi_{1}^{2}$ ^a^
High	−L	62	26.79 ± 1.22	10.14 ± 1.23	37.84	68.14	140.4^b^
	+L	21	29.43 ± 3.12	3.55 ± 1.07	12.06		
Medium	−L	142	14.39 ± 0.31	4.07 ± 0.40	28.29	83.14	87.6^b^
	+L	22	15.73 ± 0.74	0.75 ± 0.37	4.77		
Low	−L	75	6.2 ± 0.29	1.61 ± 0.27	25.91	100	
	+L	1	8	0	0		
Aggregated	−L	279	14.94 ± 0.54	4.76 ± 0.39	31.83	70.59	199.2^b^
	+L	44	22.09 ± 1.86	2.07 ± 0.58	9.36		

*Note*: Grazing cost is the proportionate reduction in % intact RU attributable to larvae.

^a^ Categorical variables = Total number of intact or lost R.U. in each category.

^b^ *p* ≪ .001.

### 3.4 Selection pressures driving the evolution of disrupted flowering in the late ecotype

**FIGURE 5:**
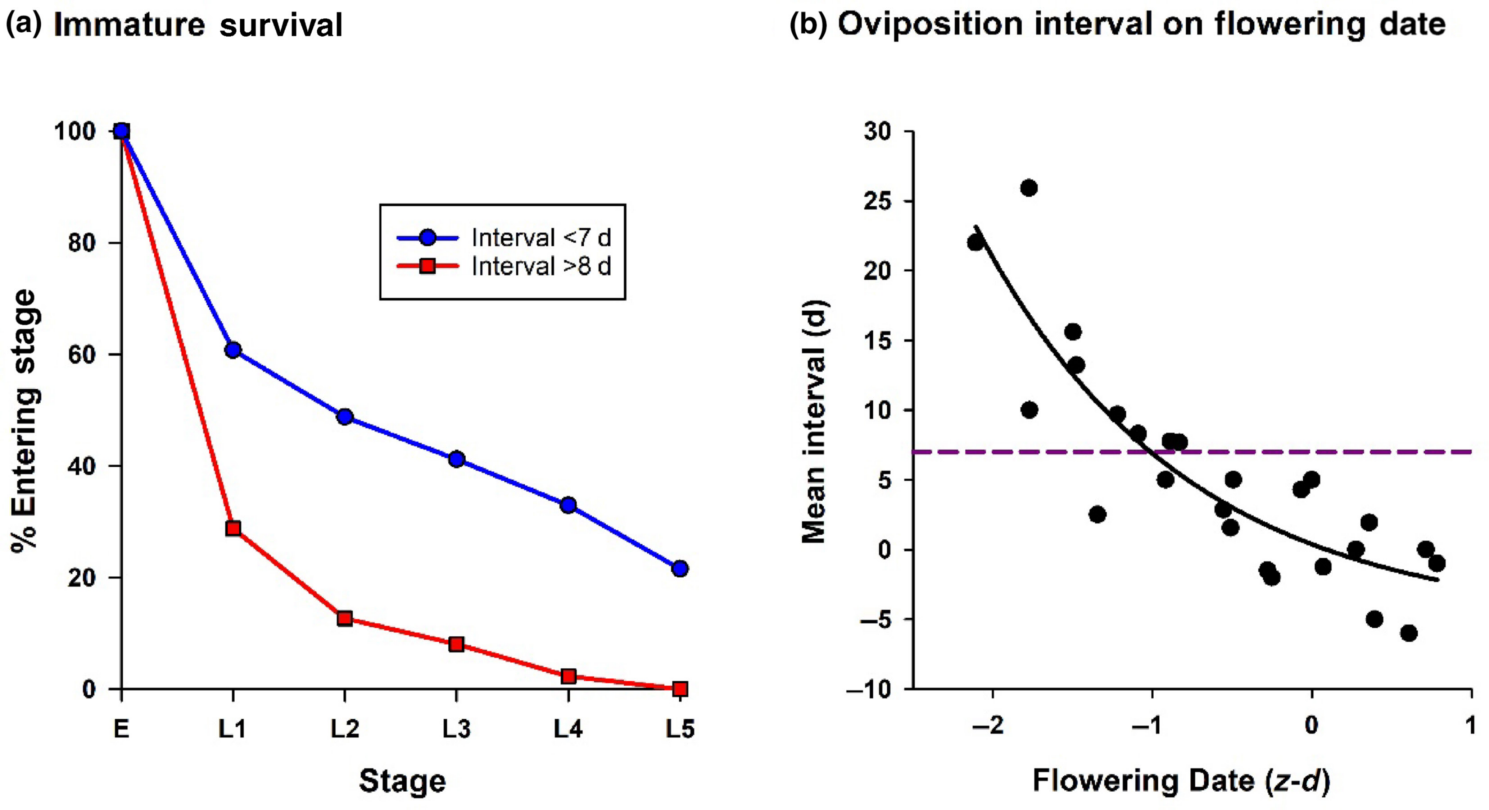
(a) Comparison between survival curves of *Anthocharis cardamines* immature stages (E = eggs, L = larval instars) on the late *Cardamine pratensis* ecotype for eggs laid within 7 days of flowering date (*N* = 158), and those laid later than 8 days after flowering date (*N* = 87). The former eggs are regarded as ‘active’, since they produced 5th instar larvae, the latter as ‘inactive’, since they did not. (b) Exponential regression of mean oviposition interval (time from flowering to egg‐laying) on high + medium fecundity late ecotype ramets versus flowering date (*z*‐transformed to standardize data from separate years in the study period: *z*‐*d* = units of SD from mean flowering date in each year). The dashed line marks the boundary between active and inactive eggs at 7 days interval. Regression equation: *y* = −5.26 + 5.64 × exp(−0.77 × *x*), *R* ^2^ = .77.

**FIGURE 6:**
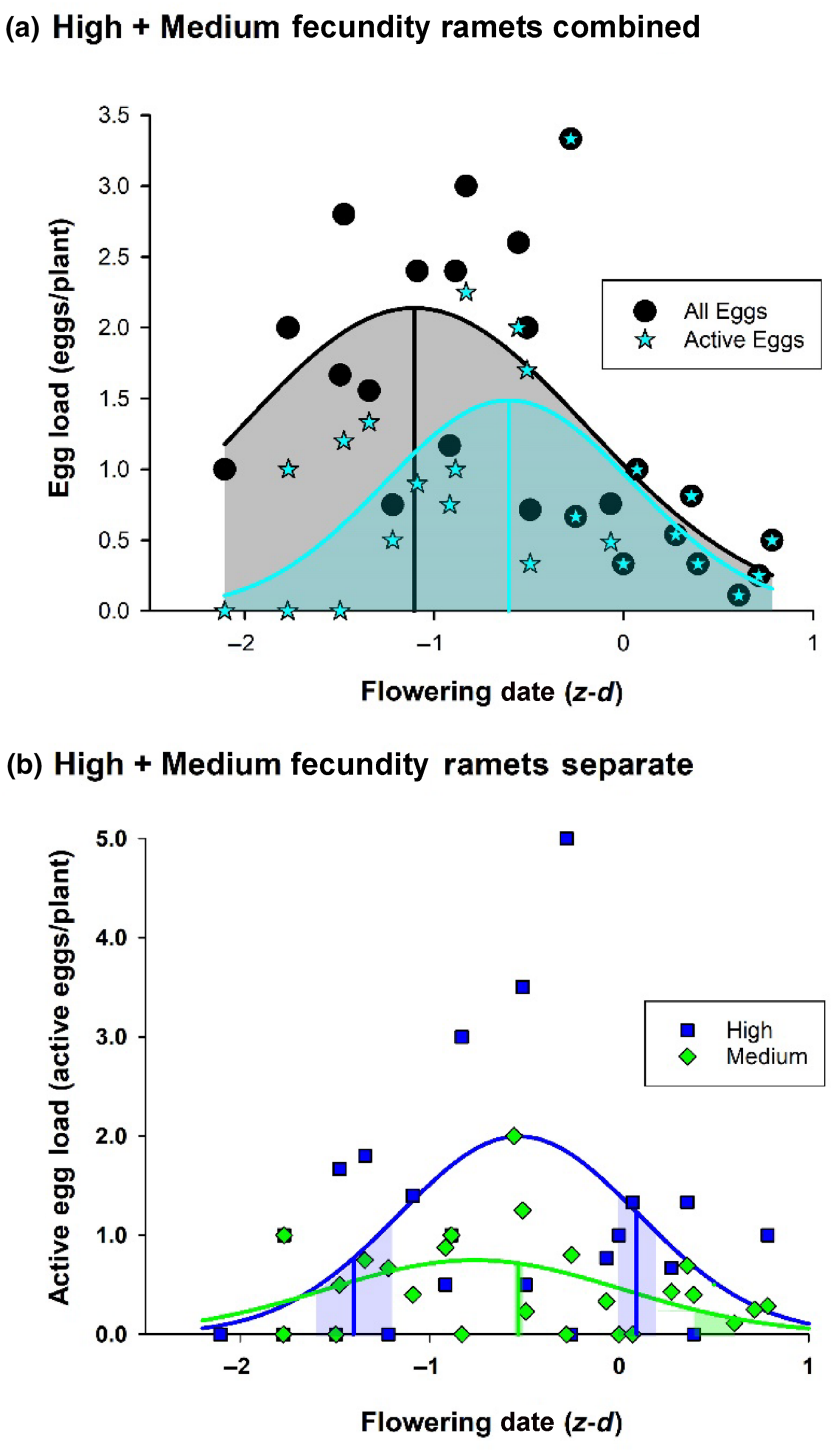
(a) Gaussian regressions of total egg load (all eggs laid per plant, black circles) and active egg load (eggs laid ≤7 days post‐flowering per plant, cyan stars) on high + medium fecundity late ecotype *Cardamine pratensis* ramets versus *z*‐transformed flowering date. The exclusion of inactive eggs has little effect in late season (overlapping data points) since most eggs laid at this time are active (region of curve below dashed line in Figure 5b); conversely, in early season many eggs are inactive (region of curve above dashed line in Figure 5b) leading to downward displacement of data points and hence reduced area under egg‐loading curve (gray/cyan shading) and translation of peak egg‐laying (black/cyan ordinates) to a later point in the season. Regression equations: *y* = 2.14 × exp(−0.5 × ((*x* + 1.10)/0.92)^2^), *R* ^2^ = .46 (total egg load); *y* = 1.49 × exp(−0.5 × ((*x* + 0.61)/0.66)^2^), *R* ^2^ = .33 (active egg load). (b) Gaussian regressions of active load on flowering date for high (blue) and medium (green) fecundity late ecotype ramets; ordinates indicate mean ± SE (shaded areas) early and late season flowering modes (when present) in each fecundity class. The data represent a decomposition of the active load curve in (a) into separate series for the two fecundity classes. Regression equations: *y* = 2.00 × exp(−0.5 × ((*x* + 0.53)/0.63)^2^), *R* ^2^ = .19 (high fecundity ramets); *y* = 0.75 × exp(−0.5 × ((*x* + 0.76)/0.78)^2^), *R* ^2^ = .13 (medium fecundity ramets).

For the late ecotype high and medium fecundity ramets, phenological escape is incomplete, since early/late emerging females have access to the two flowering peaks (Figure 4). It is therefore important to establish whether the disrupted flowering curve offers some degree of protection from the butterfly. On this ecotype eggs laid more than a week after first flowering date are ‘inactive’ in the sense that they fail to produce full grown larvae (Figure 5a; Figure S4) which inflict nearly all the damage on the plants (Table 1). Since the mean interval to egg‐laying decreases exponentially through the flight season (Figure 5b), selection on phenology must interact with early season egg deactivation. The overall effect of deactivation is to decrease the area under the egg loading curve (eggs laid per plant versus flowering date) and shift its peak to a later point in the season (Figure 6a), thereby diminishing the total damage done to the plants and moving the early season phenological refuge into the flowering period. This implies that in early flowering plants (partial) phenological escape is primarily effected through delayed time to egg‐laying (rendering eggs inactive), while in late flowering plants it is effected through reduced egg‐laying.

**FIGURE 7:**
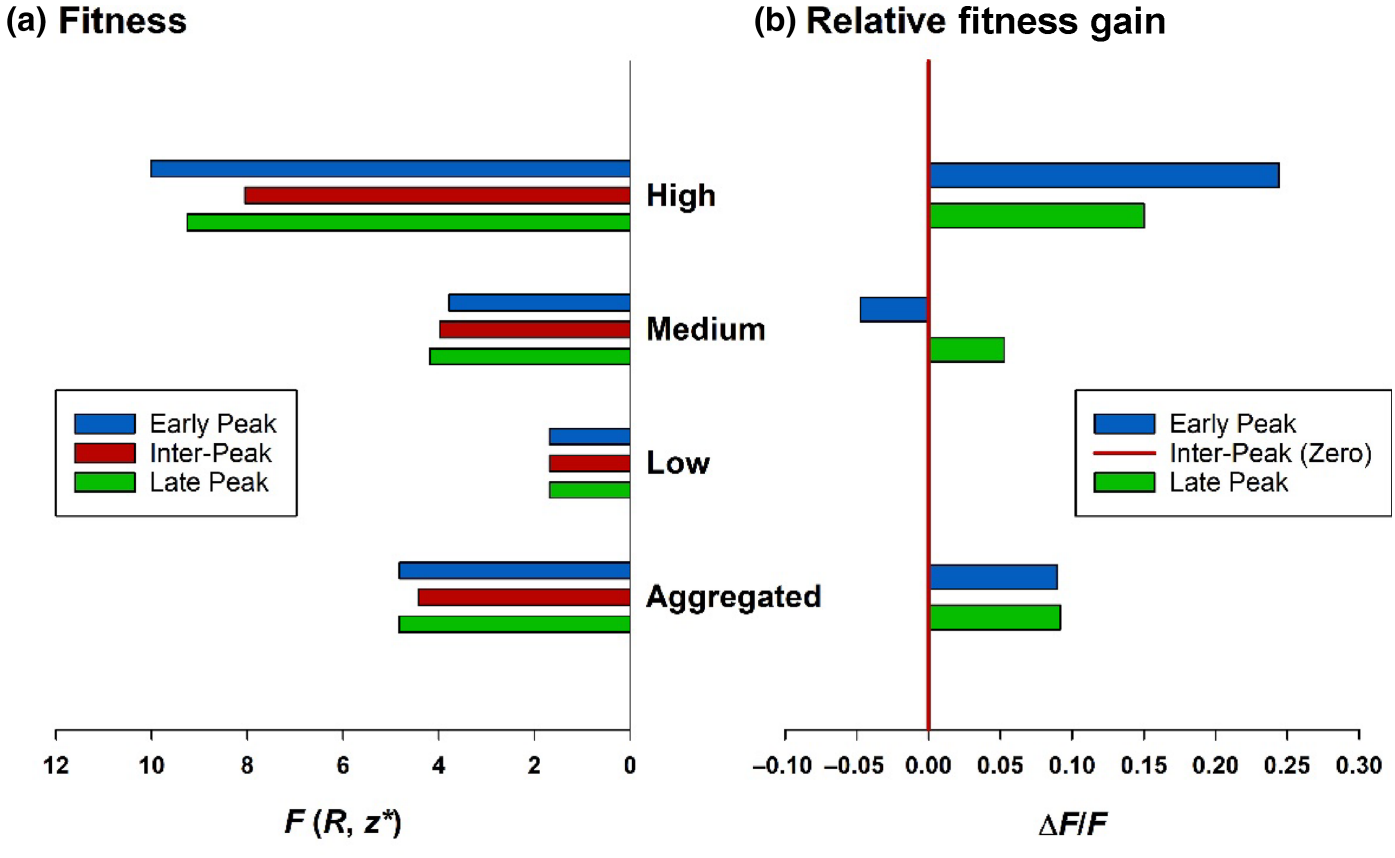
(a) Predicted early and late season peak flowering date fitness (*F* (*R*, z*)) and (b) relative fitness gain (Δ*F*/*F*) compared with flowering date fitness midway between them (inter‐peak fitness). The effect of selection operating separately on low, medium and high fecundity ramets was calculated from Equation 1, and its aggregated effect on all fecundity classes from Equation 2. In both equations, *R* was set to the average fecundity of ramets in each fecundity class (*R* = 29.8 for high, *R* = 15.4 for medium, and *R* = 6.5 for low fecundity ramets); the percentage intact seed‐pods in the presence or absence of 5th instar larvae (*I* ^+^ and *I* ^*−*^) were taken from Table 1; the active egg load and *z*‐score peak flowering dates (z*) were taken from Figure 6 (equations for *E*(*R*,*z*) given in legend or set to zero for low fecundity ramets); survival to fifth instar *L* was set to 0.22 from Figure 5a; the proportion *p*(*R*) of high and low fecundity ramets was set to 0.25 and of medium fecundity ramets to 0.5 from the definition of those fecundity classes.

Decomposition of the active load into separate curves for each fecundity class shows that high fecundity ramets are subject to more intense pressure for phenological escape (steeper wings of the Gaussian curve with higher central peak) than medium fecundity ones (Figure 6b). When fitness and relative fitness gain are calculated separately for each fecundity class (Equation 1), high fecundity ramets benefit from both early and late flowering, whereas medium fecundity ramets benefit only from late flowering (Figure 7). Therefore the early flowering peak of the medium fecundity ramets cannot be explained by phenological avoidance, but may be attributable to genetic correlation with high fecundity ramets, which are directly selected for bimodal flowering. When fitness and relative fitness gain are aggregated over all fecundity classes (Equation 2) on the assumption that each class cannot evolve independently, a bimodal curve is favored (Figure 7), indicating that bidirectional selection on high fecundity ramets is strong enough to overturn the cost of early flowering to medium fecundity plants.

## 4 DISCUSSION

Flowering phenology in *Cardamine pratensis* is subjected to strong selection pressures by the pre‐dispersal seed predator *Anthocharis cardamines*. Final instar larvae frequently (~50% of interactions) consume the entire plant prior to dehiscence (Figure S3); on average, the realized fitness (up to seed dispersal) of infested plants is reduced by ~70%. Females strongly favor larger (Figure S2) newly flowering (Figure S1) plants for oviposition; hence, flowering date is a critically important life‐history trait for high fecundity ramets. The general avoidance of overlap with the *A. cardamines* flight season of both ecotypes (Figure 4) provides strong evidence that their disrupted flowering curves are a response to phenological selection imposed by the butterfly. The late ecotype is either in an earlier phase of responding to egg‐laying pressure or the response has been arrested at an early stage due to counterbalancing selection pressures. Either way, comparison of its life history with that of the early ecotype yields mechanistic insights into the evolutionary trajectory of phenological escape with respect to plant size, oviposition preference, phenotype‐dependent selection, pre‐existing anti‐predator defense mechanisms, and the timing of highest intensity attack within the flowering period (focal point of phenological selection).

The inverse relationship between plant size and flowering date interacts with oviposition preference for large (high fecundity) ramets to determine the adaptive phenological landscape encountered by different sized plants. For the late ecotype, phenological selection on high fecundity ramets is strongly disruptive, whereas selection on medium fecundity ramets is weakly directional for late flowering (Figure 7). Hence, a phenotype‐dependent phenological response is favored. However, if the responses of the high and medium fecundity classes cannot be separated (due to strong genetic correlation between them or if fecundity is unable to act as a condition‐dependent cue capable of switching between alternative phenological strategies), a generalized flowering curve will evolve in response to differential selection pressures aggregated over all fecundity phenotypes (Figure 7), as is observed. Hence, fitness effects on high fecundity phenotypes primarily determine the phenological response, in spite of their relatively low frequency (25%) in the population, due to the steeper selection gradient operating upon them (Figure 6). The maladaptive early flowering peak of the medium fecundity ramets (Figure 7) near the (active) egg‐laying peak (Figure 6) can therefore be explained by the success of the correlated response in the high fecundity ramets. However, the bimodal response is less consistent in medium (and low) fecundity ramets, which sometimes exhibit unimodal flowering curves (Figures 2d and 3). Hence, there is a degree of phenotype‐dependency in the canalization of the response. This may reflect incomplete genetic differentiation between fecundity classes, or partial condition‐dependence in bimodal flowering. Either way, it is consistent with the early stages in the evolution of a bimodal response, which may later become specific to high fecundity phenotypes or completely generalized over all fecundity classes. While phenotypic specificity is currently favored, if the high fecundity phenotypes evolve fast enough, the first flowering peak will move far enough into early season that the associated response in the medium fecundity ramets will also be advantageous. This would favor generalized bimodal flowering, as is observed for the early ecotype, which may have evolved in this way. In conclusion, the feasibility of phenotype‐specific adaptation to differential selection pressures will shape the evolutionary trajectory of phenological escape; if the genetic correlation between traits subjected to strongly unequal selection pressures is high, and an efficient cue to decouple their respective phenologies is unavailable, the proportion of adapted phenotypes within the population may be low, and weakly maladapted phenotypes may persist in flowering refugia from which they would otherwise have been excluded.

An important counterintuitive result emerging from these studies is that the evolution of phenological escape may be facilitated rather than impeded by predator killing, since the two strategies may interact synergistically to maximize fitness. The anti‐predator defense mechanisms of the late ecotype alter its adaptive phenological landscape by reducing the number of active eggs laid and shifting the effective egg‐laying peak to a later point in the season (Figure 6a). The former effect would only dampen selection for phenological escape if the wings of the Gaussian egg‐laying curve were rendered less steep, equalizing fitness across the season, but this is not the case (Figure 6a). The anti‐predator defense mechanisms do not operate in a time‐independent way, as would be necessary if (active) egg‐laying were to be reduced to a minimal level across the entire season; instead, since the plant's defenses are only effective against eggs laid a week or more after first flowering date (Figure 5a), they are only operative in early season (Figure 5b). The fitness function is therefore time‐dependent, and the egg‐laying curve retains its original Gaussian shape (Figure 6a). The translation of the egg‐laying peak to a later point in the season moves the early season phenological refuge into the flowering period, rendering selection on the high fecundity phenotypes strongly disruptive, since the disadvantage accruing from a greater egg load on early flowering plants is overridden by the increased likelihood that oviposition will be delayed relative to flowering date (Figure 5b). It is not known whether such defense mechanisms were important in the evolution of the phenological response of the early ecotype; however, delayed oviposition is clearly important in maintaining the response since it increases the chances that the plants will outrun larval development and dehisce before seed‐pod consumption (Figure S3). In general, any benefit accruing from delayed oviposition will favor early flowering phenotypes over late flowering ones. This will always be the case when plants require time to upregulate their defenses, or when a developmental head‐start enables them to complete their life‐cycle before acquiring significant damage.

We currently have no information on the nature of the late ecotype defense mechanisms, what causes egg “deactivation,” or why it is delayed. Courtney (1981) suggested that the hardened siliquae (seed‐pods) of older plants present mechanical barriers to grazing which prevents establishment of larvae on them. This would explain the delayed onset of the defense. However, most losses resulting from late‐laid ova appear to have occurred in the egg stage (Figure 5a); while it is not impossible that some larvae hatched and then perished unobserved, the overall egg‐mortality seems too high to be entirely attributable to this cause. Additional defense mechanisms could include delayed attraction of egg predators to the plants (perhaps due to their initial inconspicuousness when not in full bloom), a delayed egg‐dropping mechanism, or delayed production/accumulation of ovicidal substances (Hilker & Fatouros, 2015). Also, the delayed production/accumulation of larval toxins or attraction of larval predators cannot be ruled out as contributing to larval mortality (Hilker & Fatouros, 2015). Regardless of the nature of a plant's anti‐predator/herbivore defense mechanisms, it is clear that any temporal dependency in their effectiveness can reshape the phenological adaptive landscape and hence redirect the evolutionary trajectory of phenological escape.

The late ecotype flowers about 25 days later on average than the early ecotype (Table S1), so that peak egg‐laying occurs at an earlier relative time during its flowering season (Figure 3). Hence, the focal point of selection changes between the two ecotypes, altering the availability of alternative phenological escape routes. Thus, the highly asymmetrical flowering curve of the early ecotype, with most plants flowering around the early season mode, can be explained by the relatively late occurrence of the focal point of selection strongly favoring escape into early season. On the other hand, the relatively early occurrence of the focal point of selection in the flowering schedule of the late ecotype exposes its high fecundity ramets to strong disruptive selection, resulting in a more equal adaptive partition of the population into early and late season. While some of these patterns may have additional or alternative explanations, such as the scarcity of pollinators in late winter or the intensity of inter‐specific competition in early summer constraining the extent to which the ecotypes can move in either temporal direction to avoid egg‐laying, it is clear that phenological escape will be strongly influenced by the initial selective configuration operating on plant populations, which may be quite different even for those flowering 25 days apart.

These insights have important implications for modeling potential evolutionary responses to unequal phenological shifts between trophic levels due to climate change. In particular, account must be taken of:‐

1. whether temporal translation of the focal point of selection alters the availability of alternative phenological escape routes;
2. how selection coefficients change over short time intervals within and adjacent to the period of overlap between interacting species;
3. the feasibility of phenotype‐specific adaptation to differential selection gradients, and the associated impact of phenotype‐dependent canalization in the early stages of phenological escape;
4. whether anti‐predator defense mechanisms impede or facilitate the evolution of phenological escape; in particular, whether temporal dependence in their effectiveness (e.g. reliance on time‐delay or a developmental head‐start) can generate phenological refugia.

A coevolutionary interaction between the phenologies of *A. cardamines* and *C. pratensis* was not detected in an analysis based solely on citizen scientist observations of first flight/flowering dates (Phillimore et al., 2012). However, a more detailed investigation with respect to plant size, flowering time, peak egg‐laying and anti‐predator defense mechanisms may be required to uncover the coevolutionary dynamics shaping the phenological interaction between these two species. If such complexity is commonplace, detailed investigation of interactions involving endangered species should be implemented at the earliest possible stage.

## AUTHOR CONTRIBUTIONS

**W. James Davies:** Conceptualization (lead); data curation (lead); formal analysis (lead); investigation (lead); methodology (lead); writing – original draft (lead); writing – review and editing (equal). **Ilik J. Saccheri:** Funding acquisition (lead); supervision (lead); writing – review and editing (equal).

## FUNDING INFORMATION

This work was funded by NERC, grant code NE/N015711/1.

## CONFLICT OF INTEREST STATEMENT

The authors have no conflict of interest to declare.

## Supporting information


Table S1.

Figure S1.

## Data Availability

The data that support the findings of this study are openly available in Dryad at https://doi.org/10.5061/dryad.v9s4mw741.

## References

[ece311330-bib-0001] Arvanitis, L. , Wiklund, C. , & Ehrlén, J. (2008). Plant ploidy level influences selection by butterfly seed predators. Oikos, 117, 1020–1025.

[ece311330-bib-0002] Chuine, I. (2010). Why does phenology drive species distribution? Philosophical Transactions of the Royal Society, B: Biological Sciences, 365, 3149–3160. 10.1098/rstb.2010.0142 PMC2981946 20819809

[ece311330-bib-0003] Colautti, R. I. , & Barrett, S. C. H. (2013). Rapid adaptation to climate facilitates range expansion of an invasive plant. Science, 342, 364–366. 24136968 10.1126/science.1242121

[ece311330-bib-0004] Courtney, S. P. (1981). Coevolution of pierid butterflies and their cruciferous foodplants III. *Anthocharis cardamines* (L.) survival, development and ovipostion on different hostplants. Oecologia, 51, 91–96. 28310315 10.1007/BF00344658

[ece311330-bib-0005] Davies, W. J. (2019). Multiple temperature effects on phenology and body size in wild butterflies predict a complex response to climate change. Ecology, 100(4), e02612. 30636278 10.1002/ecy.2612

[ece311330-bib-0006] Davies, W. J. , & Saccheri, I. J. (2013). Maintenance of body‐size variation and host range in the orange‐tip butterfly: Evidence for a trade‐off between adult life‐history traits. Ecological Entomology, 38, 49–60.

[ece311330-bib-0007] Davies, W. J. , & Saccheri, I. J. (2015). Male emergence schedule and dispersal behaviour are modified by mate availability in heterogeneous landscapes: Evidence from the orange‐tip butterfly. PeerJ, 2, e707. 10.7717/peerj.707 PMC4304852 25648908

[ece311330-bib-0008] Dempster, J. P. (1997). The role of larval food resources and adult movement in the population dynamics of the orange‐tip butterfly (*Anthocharis cardamines*). Oecologia, 111, 549–556. 28308117 10.1007/s004420050270

[ece311330-bib-0009] Dennis, R. L. H. , & Hardy, P. B. (2006). Excessive *Anthocharis cardamines* (Linnaeus, 1758) (Lepidoptera; Pieridae) egg load on the host‐plant *Cardamine pratensis* (L.) Hitt. Entomologist's Gazette, 57, 13–15.

[ece311330-bib-0010] Duggan, A. E. (1985). Pre‐dispersal seed predation by *Anthocharis cardamines* (Pieridae) in the population dynamics of the perennial *Cardamine pratensis* (Brassicaceae). Oikos, 44, 99–106.

[ece311330-bib-0011] Edger, P. P. , Heidel‐Fischer, H. M. , Bekaert, M. , Rota, J. , Glöckner, G. , Platts, A. E. , Heckel, D. G. , Der, J. P. , Wafula, E. K. , Tang, M. , Hofberger, J. A. , Smithson, A. , Hall, J. C. , Blanchette, M. , Bureau, T. E. , Wright, S. I. , dePamphilis, C. W. , Schranz, M. E. , Barker, M. S. , … Wheat, C. W. (2015). The butterfly plant arms‐race escalated by gene and genome duplications. Proceedings of the National Academy of Sciences of the United States of America, 112, 8362–8366. 26100883 10.1073/pnas.1503926112 PMC4500235

[ece311330-bib-0012] Ehrlén, J. (1996). Spatiotemporal variation in pre‐dispersal seed predation intensity. Oecologia, 108, 708–713. 28307805 10.1007/BF00329046

[ece311330-bib-0013] Ehrlén, J. (2015). Selection on flowering time in a life‐cycle context. Oikos, 124, 92–101.

[ece311330-bib-0014] Ehrlén, J. , Raabova, J. , & Dahlgren, J. P. (2015). Flowering schedule in a perennial plant; life‐history trade‐offs, seed predation, and total offspring fitness. Ecology, 96, 2280–2288. 26405752 10.1890/14-1860.1

[ece311330-bib-0015] Elzinga, J. A. , Atlan, A. , Biere, A. , Gigord, L. , Weis, A. E. , & Bernasconi, G. (2007). Time after time: Flowering phenology and biotic interactions. Trends in Ecology & Evolution, 22, 432–439. 17573151 10.1016/j.tree.2007.05.006

[ece311330-bib-0016] Fatouros, N. E. , Pineda, A. , Huigens, M. E. , Broekgaarden, C. , Shimwela, M. M. , Figueroa Candia, I. A. , Verbaarschot, P. , & Bukovinszky, T. (2014). Synergistic effects of direct and indirect defences on herbivore egg survival in a wild crucifer. Proceedings of the Royal Society B: Biological Sciences, 281, 20141254. 10.1098/rspb.2014.1254 PMC4100524 25009068

[ece311330-bib-0017] Forrest, J. , & Miller‐Rushing, A. J. (2010). Toward a synthetic understanding of the role of phenology in ecology and evolution. Philosophical Transactions of the Royal Society, B: Biological Sciences, 365, 3101–3112. 10.1098/rstb.2010.0145 PMC2981948 20819806

[ece311330-bib-0018] Franks, S. J. , Sim, S. , & Weis, A. E. (2007). Rapid evolution of flowering time by an annual plant in response to a climate fluctuation. Proceedings of the National Academy of Sciences of the United States of America, 104, 1278–1282. 17220273 10.1073/pnas.0608379104 PMC1783115

[ece311330-bib-0019] Hereford, J. (2009). A quantitative survey of local adaptation and fitness trade‐offs. The American Naturalist, 173, 579–588. 10.1086/597611 19272016

[ece311330-bib-0020] Hilker, M. , & Fatouros, N. E. (2015). Plant responses to insect egg deposition. Annual Review of Entomology, 60, 493–515. 10.1146/annurev-ento-010814-020620 25341089

[ece311330-bib-0021] Inouye, B. D. , Ehrlén, J. , & Underwood, N. (2019). Phenology as a process rather than an event: From individual reaction norms to community metrics. Ecological Monographs, 89, e01352.

[ece311330-bib-0022] Kiers, E. T. , Palmer, T. M. , Ives, A. R. , Bruno, J. F. , & Bronstein, J. L. (2010). Mutualisms in a changing world: An evolutionary perspective. Ecology Letters, 13, 1459–1474. 20955506 10.1111/j.1461-0248.2010.01538.x

[ece311330-bib-0023] Kolb, A. , Ehrlén, J. , & Eriksson, O. (2007). Ecological and evolutionary consequences of spatial and temporal variation in pre‐dispersal seed predation. Perspectives in Plant Ecology, Evolution and Systematics, 9, 79–100.

[ece311330-bib-0024] König, M. A. E. , Wiklund, C. , & Ehrlén, J. (2015). Timing of flowering and intensity of attack by a butterfly herbivore in a polyploid herb. Ecology and Evolution, 5, 1863–1872. 26140202 10.1002/ece3.1470 PMC4485967

[ece311330-bib-0025] Kőrösi, Á. , Markó, V. , Kovács‐Hostyánszki, A. , Somay, L. , Varga, Á. , Elek, Z. , Boreux, V. , Klein, A. , Földesi, R. , & Báldi, A. (2018). Climate‐induced phenological shift of apple trees has diverse effects on pollinators, herbivores and natural enemies. PeerJ, 6, e5269. 30065875 10.7717/peerj.5269 PMC6064640

[ece311330-bib-0026] Lande, R. (1979). Quantitative genetic analysis of multivariate evolution, applied to brain: Body size allometry. Evolution, 33, 402–416. 28568194 10.1111/j.1558-5646.1979.tb04694.x

[ece311330-bib-0027] Lande, R. , & Arnold, S. J. (1983). The measurement of selection on correlated characters. Evolution, 37, 1210–1226. 28556011 10.1111/j.1558-5646.1983.tb00236.x

[ece311330-bib-0028] Miller‐Rushing, A. J. , Høye, T. T. , Inouye, D. W. , & Post, E. (2010). The effects of phenological mismatches on demography. Philosophical Transactions of the Royal Society, B: Biological Sciences, 365, 3177–3186. 10.1098/rstb.2010.0148 PMC2981949 20819811

[ece311330-bib-0029] Munguia‐Rosas, M. A. , Ollerton, J. , Parra‐Tabla, V. , & De‐Nova, J. A. (2011). Meta‐analysis of phenotypic selection on flowering phenology suggests that early flowering plants are favoured. Ecology Letters, 14, 511–521. 21332621 10.1111/j.1461-0248.2011.01601.x

[ece311330-bib-0030] Nakazawa, T. , & Doi, H. (2012). A perspective on match/mismatch of phenology in community contexts. Oikos, 121, 489–495.

[ece311330-bib-0031] Ollerton, J. , & Lack, A. (1998). Relationships between flowering phenology, plant size and reproductive success in *Lotus corniculatus* (Fabaceae). Plant Ecology, 139, 35–47.

[ece311330-bib-0032] Parmesan, C. (2006). Ecological and evolutionary responses to recent climate change. Annual Review of Ecology, Evolution, and Systematics, 37, 637–669.

[ece311330-bib-0033] Pashalidou, F. G. , Lucas‐Barbosa, D. , van Loon, J. J. A. , Dicke, M. , & Fatouros, N. E. (2013). Phenotypic plasticity of plant response to herbivore eggs: Effects on resistance to caterpillars and plant development. Ecology, 94, 702–713. 23687896 10.1890/12-1561.1

[ece311330-bib-0034] Pettersson, M. W. (1994). Large plant size counteracts early seed predation during the extended flowering season of a *Silene uniflora* (Caryophyllaceae) population. Ecography, 17, 264–271.

[ece311330-bib-0035] Phillimore, A. B. , Stålhandske, S. , Smithers, R. J. , & Bernard, R. (2012). Dissecting the contributions of plasticity and local adaptation to the phenology of a butterfly and its host plants. The American Naturalist, 180, 655–670. 10.1086/667893 23070325

[ece311330-bib-0036] Rafferty, N. E. , Cara Donna, P. J. , & Bronstein, J. L. (2015). Phenological shifts and the fate of mutualisms. Oikos, 124, 14–21. 25883391 10.1111/oik.01523 PMC4396844

[ece311330-bib-0037] Sparks, T. H. , & Yates, T. J. (1997). The effect of spring temperature on the appearance dates of British butterflies 1883‐1993. Ecography, 20, 368–374.

[ece311330-bib-0038] Thompson, J. N. (2005). The geographic mosaic of coevolution. University of Chicago Press.

[ece311330-bib-0039] Wiklund, C. , & Åhrberg, C. (1978). Host plants, nectar source plants, and habitat selection of males and females of *Anthocharis cardamines* (Lepidoptera). Oikos, 31, 169–183.

