# Supplementary material for: Evolutionary trajectory of phenological escape in a flowering plant: Mechanistic insights from bidirectional avoidance of butterfly egg‐laying pressure
Source: Ecol Evol. 2024 Apr 29;14(5):e11330. doi: 10.1002/ece3.11330 (PMC11056787; doi:10.1002/ece3.11330)
Supplement: Supplementary file 1 — Table S1. [file ECE3-14-e11330-s001.docx]

## Supporting Information - Tables

**Table S1** Phenological and morphological characteristics of the early and late *C. pratensis* ecotypes in Dibbinsdale Nature Reserve 2012-2014. Fl. Date = flowering date (day of year), Fl. Dur. = flowering duration, T. Dh. = time to dehiscense (flowering date to seed-pod burst date), Fl. Ht. = flowering height, Max. Ht. = maximum height, N.A.I. = number of non-axial inflorescences, Fecundity = maximum number of buds + flowers + seed-pods. Yearly values are whole transect means ± SD; 2012-14 values are means of the three individual yearly values ± SE, mean difference values are means of the three within-year differences ± SE. Direct statistical comparison is uninformative since the large sample sizes favour significant outcomes; instead, annual means are compared using paired-sample one-tailed t-tests ([*] = 0.05<p<0.1, * = 0.01<p< 0.05, *** = p <0.001).

| Ecotype | Yr | N | Fl. Date | Fl. Dur. (d) | T. Dh. (d) | Fl. Ht. (cm) | Max. Ht. (cm) | N.A.I. | Fecundity |
| --- | --- | --- | --- | --- | --- | --- | --- | --- | --- |
| Late (1 yr mean) | 2012 | 80 | 140.0 ± 18.1  (19 May) | 20.1 ± 12.4 | 58.7 ± 13.9 | 33.6 ± 8.2 | 41.2 ± 11.7 | 0.16 ± 0.54 | 17.2 ± 10.4 |
|  | 2013 | 172 | 144.8 ± 11.8  (25 May) | 20.8 ± 8.0 | 45.3 ± 5.8 | 26.0 ± 6.8 | 33.7 ± 10.0 | 0.19 ± 0.66 | 13.8 ± 7.8 |
|  | 2014 | 71 | 128.9 ± 15.5  (9 May) | 20.0 ± 10.7 | 51.9 ± 6.0 | 33.7 ± 8.3 | 42.6 ± 11.5 | 0.42 ± 0.97 | 19.7 ± 12.0 |
| Early (1 yr mean) | 2012 | 247 | 103.6 ± 21.7  (13 April) | 21.0 ± 12.4 | 67.7 ± 6.9 | 20.1 ± 5.2 | 24.3 ± 6.7 | 0.73 ± 1.16 | 14.9 ± 11.9 |
|  | 2013 | 116 | 124.4 ± 11.5  (4 May) | 14.1 ± 7.4 | 54.7 ± 5.7 | 23.5 ± 6.5 | 27.1 ± 8.8 | 0.76 ± 1.23 | 13.6 ± 10.3 |
|  | 2014 | 124 | 106.8 ± 12.8  (17 April) | 18.5 ± 7.4 | 57.2 ± 4.3 | 28.8 ± 6.7 | 35.6 ± 9.3 | 1.05 ± 1.49 | 18.4 ± 13.0 |
| Late (3 yr mean of 1 yr means) | 2012-14 | 3 | 137.9 ± 4.7  (18 May) | 20.3 ± 0.3 | 52.0 ± 3.9 | 31.1 ± 2.6 | 39.2 ± 2.8 | 0.26 ± 0.08 | 16.9 ± 1.7 |
| Early (3 yr mean of 1 yr means) | 2012-14 | 3 | 111.6 ± 6.5  (22 April) | 17.8 ± 2.0 | 59.9 ± 4.0 | 24.1 ± 2.5 | 29.0 ± 3.4 | 0.85 ± 0.10 | 15.6 ± 1.4 |
| Mean Difference  (Late - Early) | **2012-14** | **3** | **26.3^*^ ± 5.1** | **2.4 ± 2.2** | **-7.9^*^ ± 1.3** | **7.0^[*]^ ± 3.3** | **10.2^*^ ± 3.4** | **-0.59^***^ ± 0.02** | **1.3^[*]^ ± 0.6** |
