# Supplementary material for: Evolutionary trajectory of phenological escape in a flowering plant: Mechanistic insights from bidirectional avoidance of butterfly egg‐laying pressure
Source: Ecol Evol. 2024 Apr 29;14(5):e11330. doi: 10.1002/ece3.11330 (PMC11056787; doi:10.1002/ece3.11330)
Supplement: Supplementary file 2 — Figure S1. [file ECE3-14-e11330-s002.docx]

## Supporting Information – Figures

**
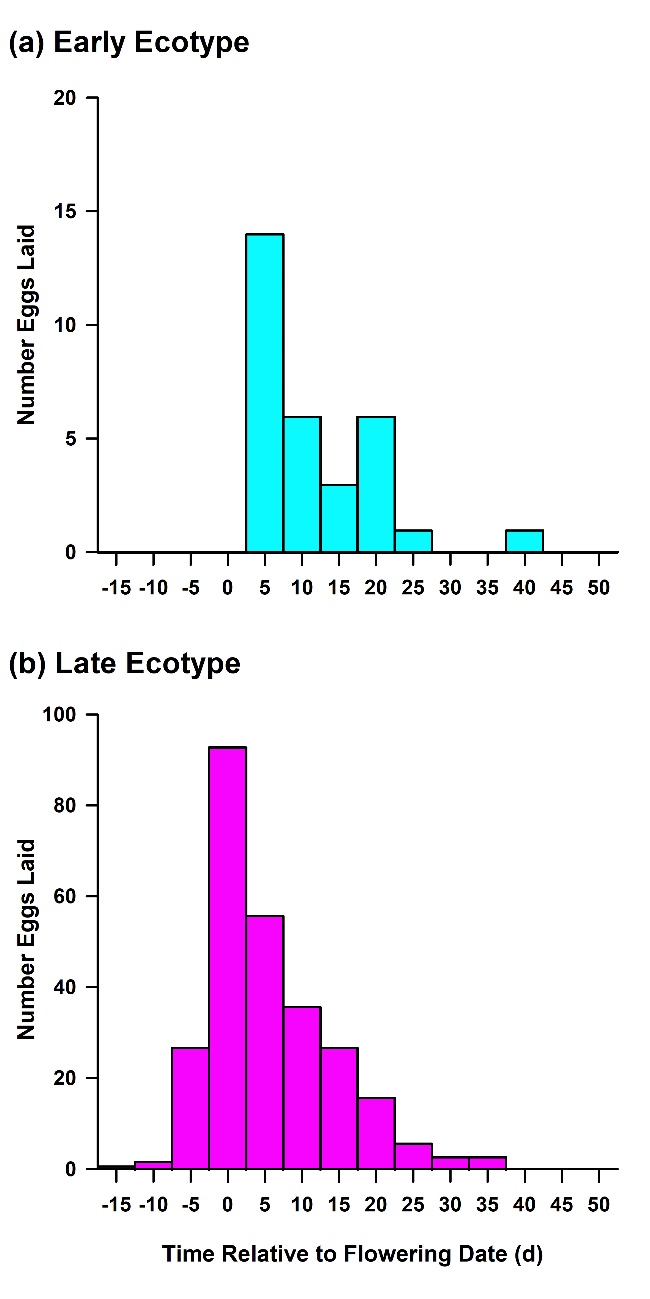
**

**Figure S1.** Relative timing of *A. cardamines* egg-laying in relation to first flowering (day zero) for (a) early ecotype (mean ± S.E. = 11.50 ± 1.44 d, N = 31), and (b) late ecotype (mean = 5.72 ± 0.53 d, N = 270) *C. pratensis* ramets in Dibbinsdale Nature Reserve 2012-2014.

**
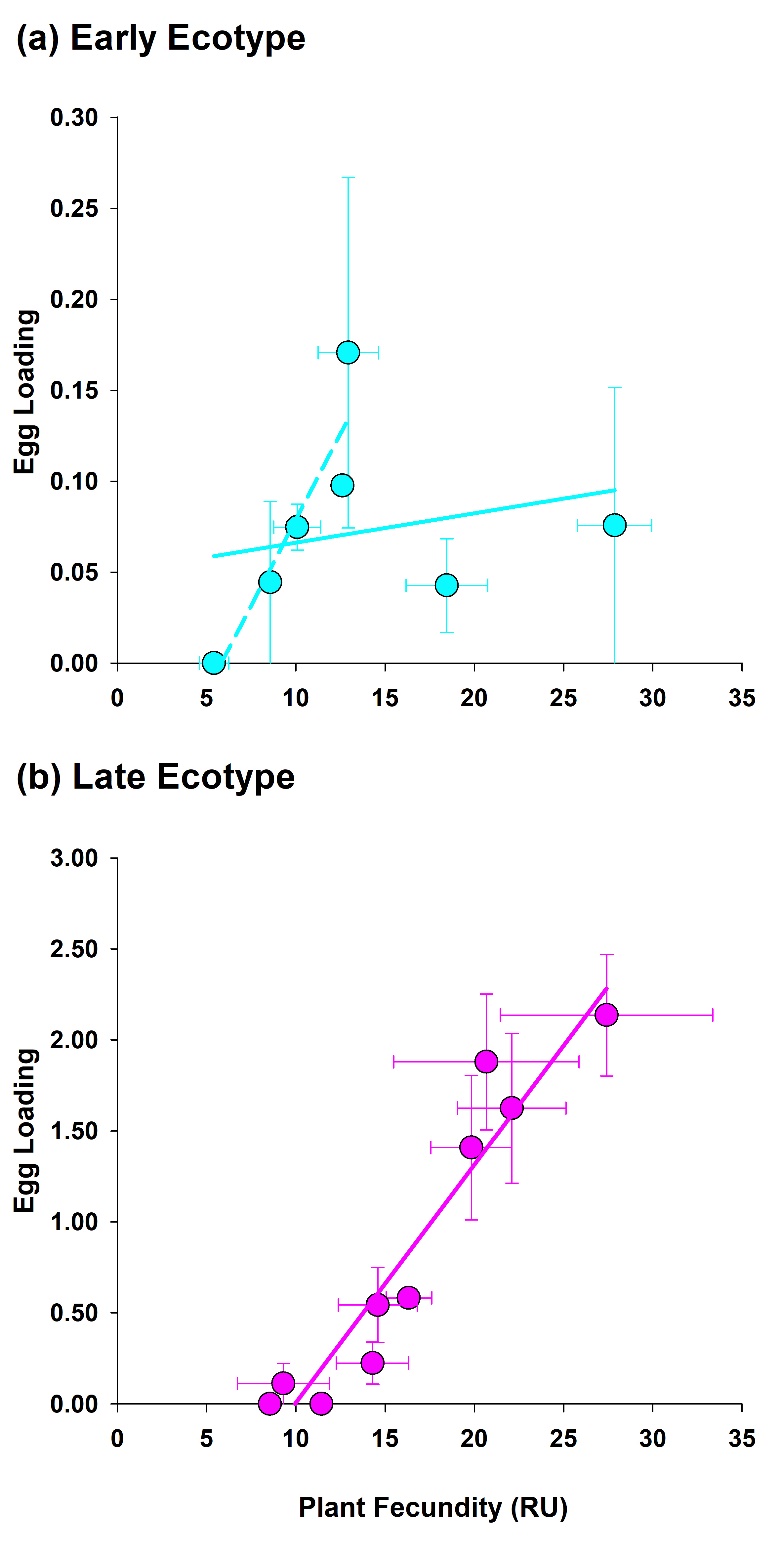
**

**Figure S2.** Regressions of egg loading (number of eggs laid per plant, E) on plant fecundity (mean number of reproductive units per plant, RU) in discrete time periods (± S.E. for inter-annual variation) for the early and late ecotypes of *C. pratensis* in Dibbinsdale Nature Reserve 2012-2014. For the early ecotype, a restricted regression limited to plants with <15 RU is also shown (dashed line). First flowering plants of both ecotypes have high mean fecundity but only those of the late ecotype are freely accessible to *A. cardamines* females, those of the early ecotype flowering before they emerge. Hence oviposition preference for high fecundity ramets of the late ecotype is disrupted for the early ecotype. Regression equations: E = 0.1307*RU - 1.2994, R^2^ = 0.91, p = 0.00002 (late ecotype); E = 0.0016*RU + 0.0502, R^2^ = 0.05, p = 0.63 (early ecotype); E = 0.0189*RU -0.1101, R^2^ = 0.92, p = 0.03 (restricted early ecotype).


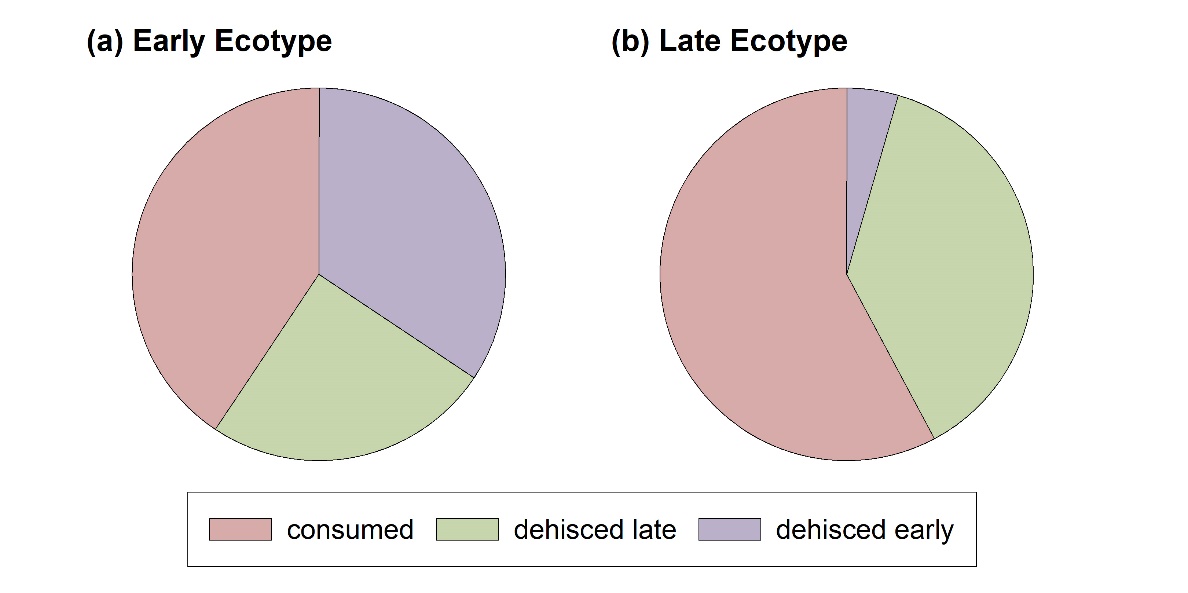


**Figure S3.** Pie charts for the relative proportion of *C. pratensis* plants bearing final instar *A. cardamines* larvae which were entirely consumed before dehiscence ("consumed"), dehisced after the completion of larval growth ("dehisced late"), or dehisced before the completion of larval growth ("dehisced early"), hence reducing seed loss. (a) early ecotype (2017-18), n = 32; (b) late ecotype (2012-14), n = 45.

**
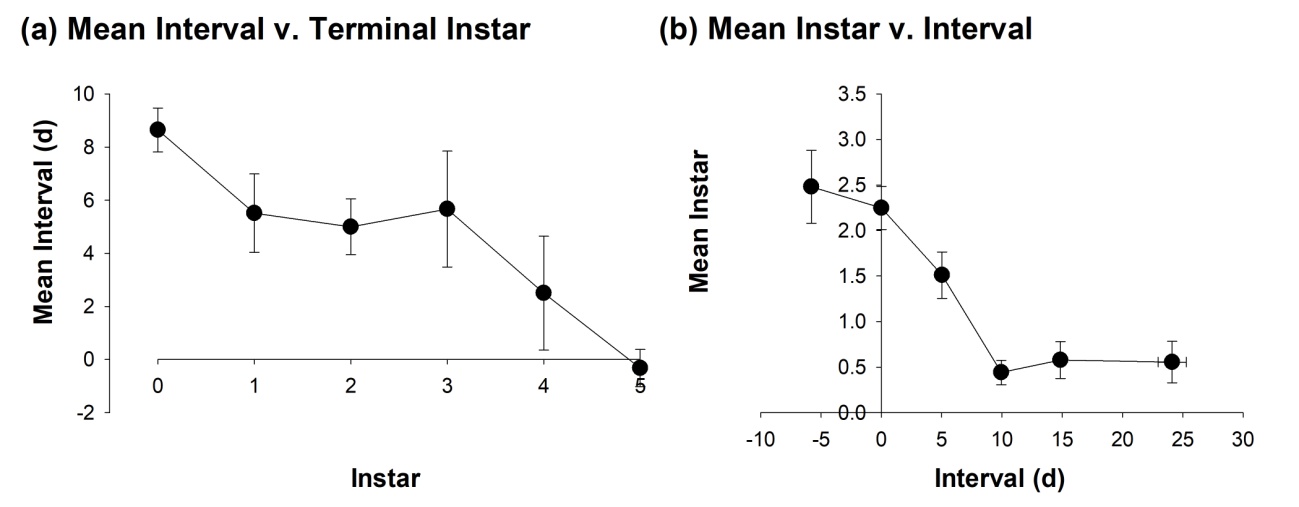
**

**Figure S4.** Relationship between larval survivorship and oviposition interval on the late *C. pratensis* ecotype. The same dataset expressed in alternate ways to highlight the strong selection pressure on *A. cardamines* females to oviposit near the time of first flowering, and the pressure on *C. pratensis* plants to extend the interval beyond 5 days to minimize damage. (a) Mean ovipostion interval (time elapsed between flowering and egg laying) for each instar reached by *A. cardamines* immature stages (0 = egg stage, 1-5 = larval instars). Each data point is restricted to specimens last observed in that instar; data do not include specimens subsequently observed in later instars. The fate of larvae after the 5th instar is unknown, since they leave the hostplant to pupate. (b) Mean instar reached against oviposition interval (averaged in discrete bins within the continuous range of observed intervals). All values ± SE.
